# Microassembly of Heterogeneous Materials using Transfer Printing and Thermal Processing

**DOI:** 10.1038/srep29925

**Published:** 2016-07-18

**Authors:** Hohyun Keum, Zining Yang, Kewen Han, Drew E. Handler, Thong Nhu Nguyen, Jose Schutt-Aine, Gaurav Bahl, Seok Kim

**Affiliations:** 1 Department of Mechanical Science and Engineering, University of Illinois at Urbana-Champaign, Urbana, Illinois 61801, USA; 2 Department of Electrical and Computer Engineering, University of Illinois at Urbana-Champaign, Urbana, Illinois 61801, USA

## Abstract

Enabling unique architectures and functionalities of microsystems for numerous applications in electronics, photonics and other areas often requires microassembly of separately prepared heterogeneous materials instead of monolithic microfabrication. However, microassembly of dissimilar materials while ensuring high structural integrity has been challenging in the context of deterministic transferring and joining of materials at the microscale where surface adhesion is far more dominant than body weight. Here we present an approach to assembling microsystems with microscale building blocks of four disparate classes of device-grade materials including semiconductors, metals, dielectrics, and polymers. This approach uniquely utilizes reversible adhesion-based transfer printing for material transferring and thermal processing for material joining at the microscale. The interfacial joining characteristics between materials assembled by this approach are systematically investigated upon different joining mechanisms using blister tests. The device level capabilities of this approach are further demonstrated through assembling and testing of a microtoroid resonator and a radio frequency (RF) microelectromechanical systems (MEMS) switch that involve optical and electrical functionalities with mechanical motion. This work opens up a unique route towards 3D heterogeneous material integration to fabricate microsystems.

While monolithic microfabrication has been quite successful in the manufacturing of microsystems such as integrated circuits (IC) and microelectromechanical systems (MEMS)^1^,^2^, continued innovation towards three dimensional (3D) architectures and heterogeneous integration has been limited, which would otherwise enable improvements in performance and novel functionalities of microsystems. Associated challenges originate from layer-by-layer thin film processing on a single substrate and dissimilar nature of materials that may need different techniques to process. Consequently, 3D heterogeneous integration often requires independent fabrication of constituents followed by microassembly rather than monolithic microfabrication. In this context, transfer printing^3^,^4^ has emerged as a method that utilizes highly reversible surface adhesion of a polymeric stamp to deterministically transfer microscale solid objects called “inks”. The ability to transfer inks from a donor substrate where inks are grown and processed to a receiving substrate where inks are finally assembled reduces the complexity of manufacturing processes regarding heterogeneous material integration. Furthermore, previously reported micro-masonry^5^ which relies on transfer printing demonstrates that after proper thermal processing, direct bonding between transferred silicon inks can be achieved, which may be sufficiently strong to produce various MEMS devices^6^,^7^,^8^. However, limited assembling material classes and quantitatively unknown interfacial characteristics between joined inks suppress broader adaptation of this transfer printing-based microassembly.

Here, we extend micro-masonry to an approach to assembling microsystems with four disparate classes of device-grade materials including Si (semiconductor), SiO_2_ (dielectric), Au (metal), and epoxy-based SU8 (polymer) at the microscale. We refer to this approach as ‘micro-Lego’ due to the similarities to the commercial product, Lego, in the aspects of stacking and joining of different types of building blocks while at different scales. Four different materials are processed into inks, assembled into spatially organized 3D architectures via reversible adhesion-based transfer printing followed by thermal processing-based material joining. Depending on assembled material pairs, diverse joining mechanisms including fusion^9^,^10^, eutectic^11^, and adhesive^12^ bonding are explored at the microscale. The joining strength for each mechanism is examined utilizing blister tests^13^,^14^ not only for systematic comparison of joining characteristics of four different assembled material pairs but also for quantitative validation of the structural integrity enabled by micro-Lego. In an effort to demonstrate 3D heterogeneous integration capabilities of micro-Lego, various 3D microstructures, a microtoroid resonator and a radio frequency (RF) MEMS switch are assembled with Si, SiO_2_, Au and SU8. Furthermore, electrical connection between constituents on different steps of the RF MEMS switch is fulfilled to highlight 3D interconnection by micro-Lego. These 3D heterogeneous architectures are extremely challenging or inaccessible to reproduce via conventional monolithic microfabrication or other microassembly techniques such as those based on robotic pick-and-place^15^ and fluidic assembly^16^.

## Results

### Procedure of micro-Lego

**Figure 1:**
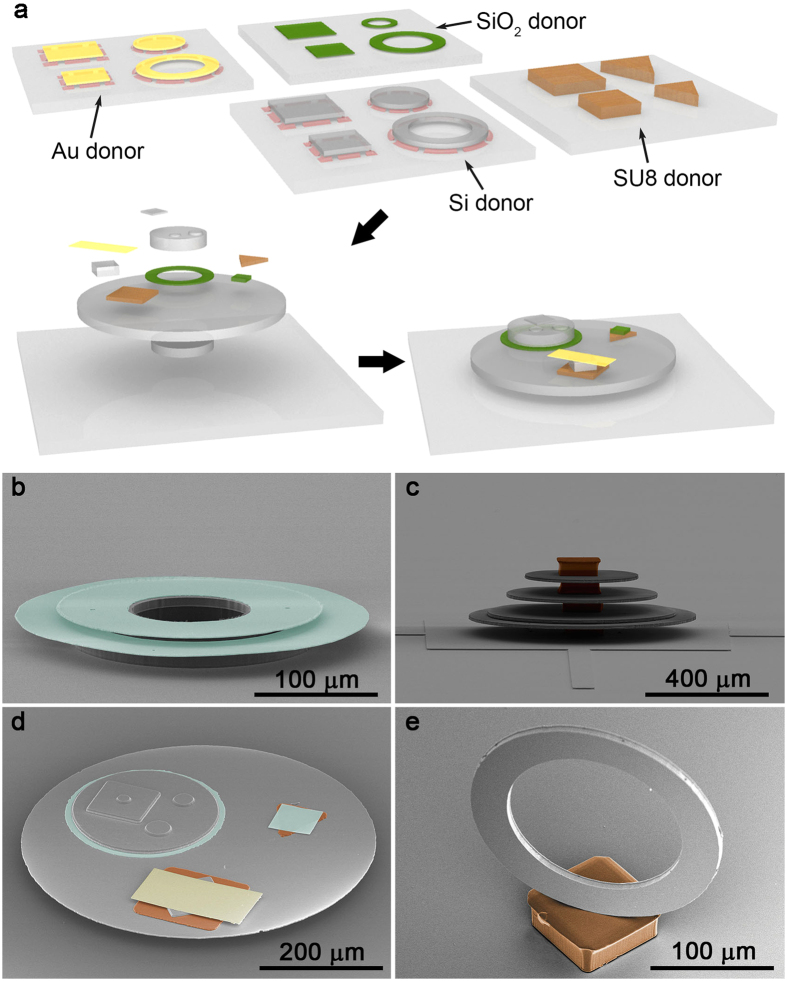
Schematic illustration of micro-Lego and SEM images of representative assembled microstructures. Scanning electron microscopy (SEM) images are colored with green, yellow, brown, and uncolored to differentiate SiO_2_, Au, SU8, and Si, respectively. **(a)** Four different inks are prepared on four different donor substrates (Supplementary Fig. 1 through Supplementary Fig. 4). Different inks are individually transferred onto target sites on a receiving substrate and joined together by thermal processing. Detailed procedure of micro-Lego is presented in Supplementary Fig. 6. **(b)** Double layer Si rings and SiO_2_ discs. **(c)** Multiple layer Si discs and SU8 blocks. **(d)** Microstructure composed of Si, SiO_2_, Au, and SU8 inks. **(e)** A vertically aligned Si ring joined on a SU8 block.

Figure 1(a) represents the procedure of micro-Lego that can be divided into three sequential steps: preparation, transferring, and joining of inks. The ink materials addressed in this work are single crystalline Si, thermally grown SiO_2_, sputter deposited Au and lithographically patterned SU8, and they are processed into ink arrays such that they are easily retrieved from donor substrates during transfer printing (Supplementary Fig. 1 through Supplementary Fig. 4). An ink prepared on a donor substrate is transferred onto a target area of a receiving substrate utilizing a microtip stamp as described in Supplementary Fig. 5, Supplementary Fig. 6 and elsewhere^4^. The receiving substrate with the transferred ink is thermally processed subsequently to join the ink and a surface where it is placed by fusion^9^,^10^, eutectic^11^ or adhesive^12^ bonding in an appropriate condition (Supplementary Table 1). This transferring and joining cycle is repeated until an anticipated 3D architecture is achieved as presented in Fig. 1(b–e). The scanning electron microscopy (SEM) images are colored to emphasize the assembly of different classes of materials, i.e., Si (uncolored), SiO_2_ (green), Au (yellow), and SU8 (brown) into the structures. It is worthwhile to note that the temperature of individual joining process should be considered when the sequence of assembling materials is set since materials such as SU8 or Au may not withstand the high temperature for Si-Si or Si-SiO_2_ fusion bonding. While 3D assembled structures demonstrated here do not exhibit device functionalities, these 3D structures realized from 2D inks at the microscale present the unparalleled heterogeneous material assembly capabilities of micro-Lego that can further be exploited in countless applications.

### Characterization of interfacial joining strength enabled by micro-Lego

**Figure 2:**
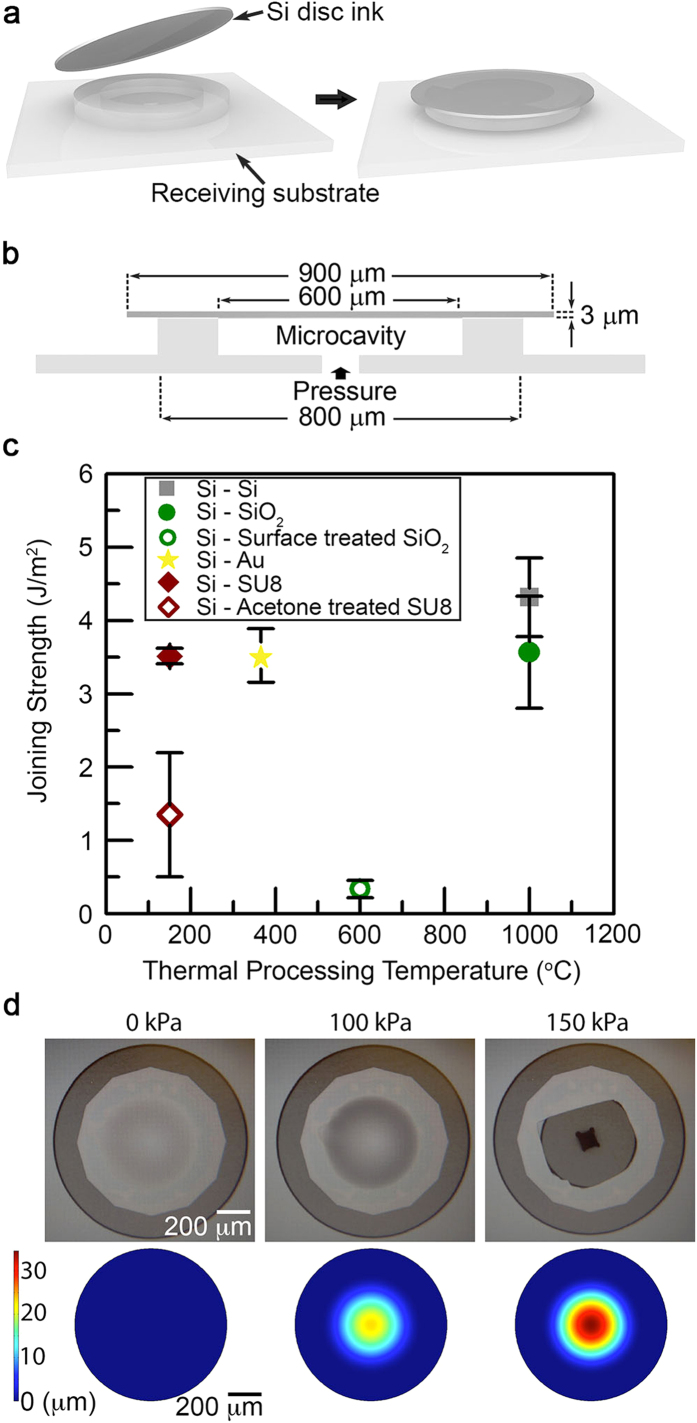
A blister test specimen and results of measured joining strength. **(a)** Schematics of a Si-Si blister test specimen assembled via micro-Lego. A Si disc ink, separately prepared on a donor substrate, is transferred onto a receiving substrate and joined for hermetic sealing through Si-Si fusion bonding. Si, SiO_2_, Au and SU8 receiving substrate fabrication procedures are detailed in Supplementary Fig. 7. **(b)** Cross section view of the assembled specimen. **(c)** Comprehensive data of joining strength with respect to material pairs and thermal processing temperatures obtained through blister tests. **(d)** Representative optical microscope images and finite element analysis (FEA) results for a Si-Si blister test specimen at three pressure states.

A blister test, which has been successfully utilized to characterize the adhesion of thin films formed on Si substrates^13^,^14^, is adopted for measuring the joining strength at Si-Si, Si-SiO_2_, Si-Au and Si-SU8 interfaces created through micro-Lego. The procedure to make a Si specimen for a blister test and its dimensions are depicted in Fig. 2(a,b). Full description of blister test specimen fabrication, joining conditions and testing procedures are in Materials and Methods in Supplementary Information, Supplementary Fig. 7 through Supplementary Fig. 9 and Supplementary Table 2. The pressure inside hermetically sealed microcavity increases in a controlled manner using a syringe pump, which induces the delamination of a Si ink from the rim structure on a receiving substrate at critical pressure that satisfies Griffith’s fracture criterion^13^:
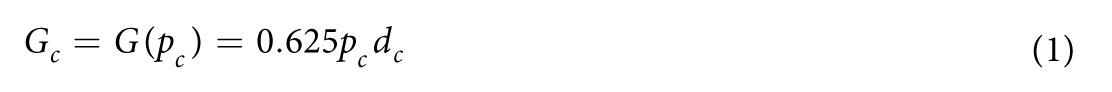
where *G*_*c*_ is a material property termed critical energy release rate or toughness, which indicates the material’s resistance to fracture along any given path. Provided that a Si ink delaminates along the joining interface, the corresponding *G*_*c*_ indicates the toughness of the joining. The energy release rate *G*, on the other hand, is a loading parameter indicating the driving force for fracture. For the specimen geometry here, *G* is simply a function of the applied pressure *p*_*c*_ and the central deflection of the Si ink *d*_*c*_^13^. Finite element analysis (FEA) is conducted to determine *d*_*c*_ that is a function of measured *p*_*c*_, specimen dimensions and material properties. At the moment when the Si ink delamination occurs, *G* reaches the toughness *G*_*c*_. Three specimens are tested per each joining material pair and the resultant *G*_*c*_ are plotted in Fig. 2(c) with respect to their thermal processing temperatures. Two different joining conditions are investigated in both Si-SiO_2_ and Si-SU8 pairs to compare the optimal and as conducted cases (refer to Supplementary Table 2). Figure 2(d) provides optical images of an assembled Si ink upon pressuring and FEA results where the ink is ruptured prior to delamination from the underneath Si rim, indicating that measured value for a Si-Si pair in Fig. 2(c) is the lower bound of the actual joining strength.

### Microtoroid resonator assembled via micro-Lego

**Figure 3:**
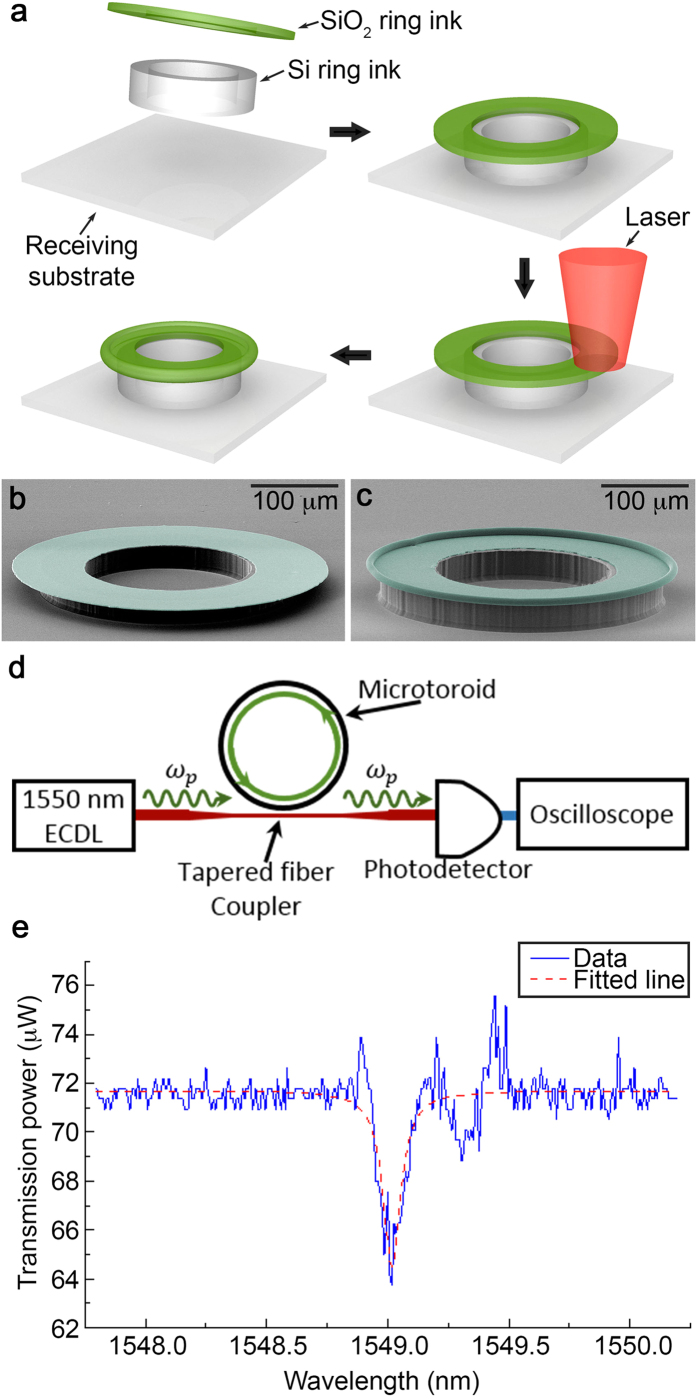
Fabrication and testing of a microtoroid resonator. **(a)** Schematics of a microtoroid resonator fabrication. Through micro-Lego, Si ring and SiO_2_ disc inks are assembled. The assembled structure is illuminated with 10.6 μm wavelength light from a 10 W CO_2_ laser to induce material reflow at the outer circumference of the SiO_2_ disc, resulting in microtoroid shaped whispering-gallery photonic resonator. (**b,c**) SEM images of the assembled structure before and after lasing. These images are colored to provide distinction between different materials. Untouched and green color represent Si and SiO_2_, respectively. **(d)** Laser light is coupled into a microtoroid resonator through a tapered optical fiber. Forward propagating light in the fiber is collected at a photodetector and analyzed by an oscilloscope. **(e)** Power transmission in the fiber is measured with respect to wavelength, and shows the characteristic resonance dip associated with the microtoroid resonance. Lorentzian curve fitting indicates Q-factor of 1.7 × 10^4^.

To demonstrate the device level capability of micro-Lego, a microtoroid shaped photonic whispering-gallery resonator (WGR) is assembled and tested. Thanks to their extremely high optical Q-factors, WGRs find extensive use in nonlinear optics^17^,^18^,^19^,^20^. Ultra-high-Q silica (SiO_2_) microresonator of toroidal and wedge-disk geometries are typically fabricated using a combination of chemical etching and laser-reflow^21^. This process involves undercutting of a silica disk using XeF_2_ isotropic etch. To obtain microtoroids, the undercut is followed by physical reflow under high-power CO_2_ laser illumination (10.6 μm wavelength). The key challenge that persists with these methods is that ultra-high-Q silica resonators cannot be co-integrated with other planar photonic and electronic devices on a silicon substrate, since the resulting silicon substrate is nonplanar and frequently pitted. Micro-Lego, on the other hand, is capable of fabricating this geometry without any need for undercut as it merely joins pre-fabricated Si and SiO_2_ ring-shaped inks individually. Additionally, more complex multi-layered WGR geometries are now permissible (Fig. 1(b)) which are not practical by conventional microfabrication. Figure 3(a) shows a process flow for producing a SiO_2_ microtoroid through micro-Lego and Fig. 3(b,c) show SEM images of the device before and after reflow of the disk. This fabricated microtoroid resonator is tested via tapered fiber-coupling^22^ as depicted in Fig. 3(d) in the test setup (Supplementary Fig. 10). Optical transmission (Fig. 3(e)) measured through the waveguide shows a characteristic Lorentzian shaped 0.087 nm wide optical resonance of the WGR at 1549 nm and the extracted Q-factor is about 1.7 × 10^4^.

### RF MEMS switch assembled via micro-Lego

**Figure 4:**
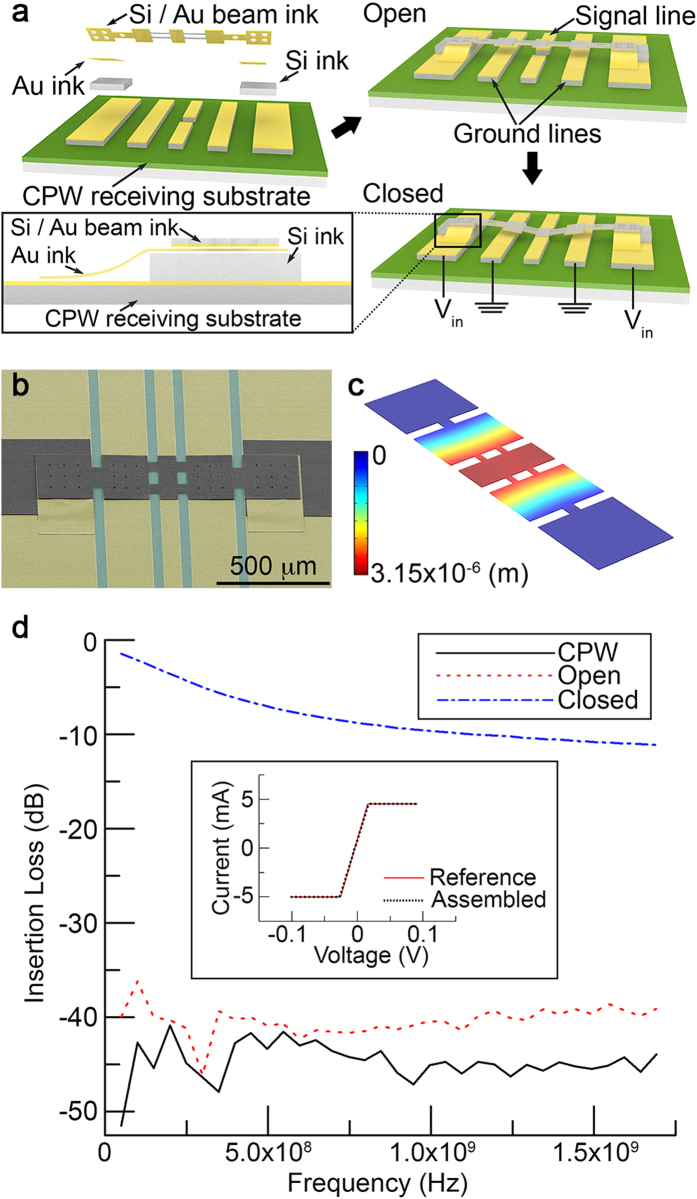
Fabrication and testing of a series contact type RF MEMS switch. **(a)** Schematics of a radio frequency (RF) microelectromechanical systems (MEMS) switch assembled via micro-Lego. The suspended beam on a coplanar waveguide (CPW) substrate deflects upon biasing resulting in physical contact with the center signal line. An inset cross-sectional illustration highlights 3D interconnection using an Au ink assembled on two different steps. **(b)** A SEM image of the assembled RF MEMS switch. The image is colored to provide distinction between different materials. Untouched, green and yellow colors represent Si, SiO_2_ and Au, respectively. **(c)** FEA simulation of the mechanical deflection of the suspended beam. Upon 25 V, the central region of the beam deflects approximately 3.6 μm allowing for physical contact with the center signal line. **(d)** Measured insertion loss data in CPW, open and closed states. The inset represents I-V curve for an assembled Au ink on an Au surface. (Supplementary Fig. 13).

Micro-Lego is further utilized to assemble a series contact type RF MEMS switch. Common RF MEMS switches require 3D suspended architectures for electromechanical performances, which is complicated to produce using microfabrication due to limited material choice for a sacrificial layer as well as stiction during wet processes^23^. To mitigate these manufacturing challenges, flip-chip transfer techniques^24^ are proposed and those approaches typically allow a single transfer of a complex part to fabricate a device. On the contrary, micro-Lego consecutively transfers and joins individual components of a device in dry conditions, which significantly simplifies the manufacturing procedure and grants more freedom in device design compared to microfabrication and other transfer techniques^24^,^25^. Figure 4(a) exhibits the assembly procedure of a series contact type RF MEMS switch. Once the device is formed, voltage bias is applied between a suspended beam and two ground lines on each side of the center signal line, resulting in the suspended beam to deflect down via the electrostatic force. It is noted that the assembled beam ink consists of a thin Au layer under a Si backbone. To electrically connect outermost lines and the thin Au layer of the beam, which are located on different steps, additional Au inks are transferred and joined in between via Au-Au cold welding^26^ as depicted in the inset illustration of Fig. 4(a). Detailed device design and assembly procedure are included in Supplementary Fig. 11. Figure 4(b,c) represent a colored SEM image of the fully assembled RF MEMS switch and FEA simulation of the beam deflection, respectively. Insertion loss of the RF MEMS switch obtained using the test setup (Supplementary Fig. 12) is shown in Fig. 4(d). In open state, the collected data show high insertion loss similar to that of coplanar waveguide (CPW) substrate due to the disconnected center signal line. The insertion loss is, however, significantly reduced upon biasing owing to the fully collapsed Si/Au beam that makes a physical contact with the disconnected signal line and induces electrical connection. Inset plot of Fig. 4(d) exhibits the current-voltage (I-V) curve representing electrical connection through a transferred and joined Au ink to further validate that Au inks do not alter the electrical performance when they are assembled by micro-Lego (Supplementary Fig. 13).

## Discussion

Micro-Lego relies on thermal processing for material joining similar to conventional wafer bonding techniques^27^ that commonly involve external forces to maintain an intimate contact between wafers. However, individual joining process of micro-Lego does not require such external forces during thermal processing since the typical assembling inks are significantly smaller than wafers such that intermolecular force between inks is strong enough to maintain sufficient surface contact. Those domineering intermolecular forces presumably originate from reduced defects in small contact area compared to wafer-scale area. To our knowledge, there has been no report of characterizing these joining methods using assembled microstructures. Thus, the experimental assessment of the interfacial joining strength between assembled inks can not only ensure the robustness of structures constructed through micro-Lego but also endow the capability of comprehensive comparison between various mechanisms to join different materials at the microscale. Remarkably, all obtained data for the four material pairs plotted in Fig. 2(c) are similar to or higher than the toughness data for silicon wafer bonding measured elsewhere^9^,^10^,^11^ although each joining here is achieved without external forces during thermal processing as opposed to wafer bonding techniques. Yet, it is worthwhile to mention that for the Si-Au blister test specimen, ~150 kPa is applied during thermal processing to form hermetic sealing in the microcavity. Without the pressure, the bonding is formed at localized regions, which invalidates the application of Equation 1. Nevertheless, all other Au inks assembled through micro-Lego in this work are joined without such external pressure. Therefore, the measured joining strength at Si-Au interface shown in Fig. 2(c) is the upper bound of the actual joining strength.

While thermal processing conditions for material joining in micro-Lego are adopted from conventional wafer bonding techniques, optimal wafer-scale thermal processing conditions are not necessarily exactly replicated at the microscale when implementing micro-Lego processes. For example, an assembled Si-SiO_2_ structure may fail to retain its original structure after thermal processing because of different thermal expansion coefficient between Si and SiO_2_. In this case, relatively low temperature (600 °C) in conjunction with oxygen plasma surface activation may be exercised in micro-Lego. To reproduce such a process for a blister test specimen, a SiO_2_ coated receiving substrate is activated using oxygen plasma followed by transfer printing and thermal processing. The test results yield 0.3 J/m^2^, which is lower than for Si-SiO_2_ pair joined at the temperature of 1000 °C, but still on par with other known wafer-scale joining strength^10^. It has been reported that surface activation on SiO_2_ surface results in slightly higher joining strength^28^ which leads to marginally reduced joining strength for the occasions where Si surface is activated prior to joining. Similarly, joining strength between Si and acetone treated SU8 is separately studied since SU8 inks for micro-Lego are prepared by releasing SU8 inks in acetone bath. As expected, the acetone treatment on SU8 reduces the joining strength in comparison with unadulterated Si-SU8 interface, but it is still on the same order of magnitude with all other obtained joining strength data.

This work was intended to develop a novel transfer printing and thermal process based microassembly technique to enable highly organized delicate microscale 3D architectures with high structural integrity that are challenging otherwise. The joining strength data obtained through blister tests (Fig. 2(c)) as well as electrical conductance between Au – Au (Inset plot in Fig. 4(d)) and Au-Si^29^ strongly support the device level capabilities of the micro-Lego technique. While the experimentally obtained Q-factor and insertion loss from the microtoroid resonator and the RF MEMS switch presented in this work display their functionalities, they do not outperform their state of the art counterparts. For example, the Q-factor of the microtoroid resonator fails to compete with other advanced microtoroid resonators^30^. This is the result of non-uniform reflow as well as device design that focused on manufacturing convenience rather than performance optimization. With improved processes and optimized design, micro-Lego-assembled devices could potentially achieve performances on par with other state of the art counterparts.

## Conclusion

In summary, microassembly of four different classes of materials including Si, SiO_2_, Au, and SU-8 is presented here. The method employs reversible adhesion-based transfer printing and thermal processing-based material joining. The interfacial joining characteristics between dissimilar materials are quantitatively studied through blister tests to validate the structural integrity of assembled structures and devices. Utilizing this approach, a few microsystems, including a microtoroid resonator and a RF MEMS switch, are fabricated and their device performances are examined. This work finds a unique way for 3D heterogeneous microsystems, with relevance not only to MEMS but also to electronics, photonics, metamaterials, and other fields.

## Methods

### Microassembly procedure

Si, Au, SiO_2_ and SU8 inks are prepared on individual donor substrates as described in Supplementary Fig. 1 through Supplementary Fig. 4. A microtip stamp made of PDMS (Supplementary Fig. 5) is brought to contact with an ink with high preload such that all microtips are fully collapsed. Rapid retreival of the microtip stamp allows the ink to be separated from the donor substrate and adhere to the microtip stamp. Once the preload is removed, the stamp restores to its original microtip configuration, which results in minimal adhesion between the retrieved ink and the stamp due to the reduced contact area. Subsequently, the stamp with the ink is delivered to a target area on a receiving substrate and brought to contact with the substrate with minimal preload. The stamp is then separated from the substrate at low speed, which leaves the ink on the target area due to the stronger intermolecular interaction between the receiving substrate and the ink (Supplementary Fig. 6). Following transfer printing of an ink is thermal processing (Supplementary Table 1) to join the ink and the substrate.

### Microassembly of blister test specimens

Receiving substrates with rims covered or fomred by four different materials are made as depicted in Supplementary Fig. 7. On a separate donor substrate, Si disc inks are fabricated and these inks are assembled on the receiving substrates as illustrated in Supplementary Fig. 8. Supplementary Fig. 9 describes the test setup where the hermetically sealed specimens are pressurized until Si disc inks are delaminated or ruptured. The joining conditions are provided in Supplementary Table 2.

### Microassembly and testing of a microtoroid resonator

Si (20 μm thick) ring and SiO_2_ (1 μm thick) disc inks are brought and joined together on a silicon receiving substrate. The edge of the SiO_2_ disk ink is then reflowed with CO_2_ laser (10.6 μm wavelength) illumination into the requisite microtoroid geometry. The SiO_2_ microtoroid resonator is tested by measuring optical transmission, using a tunable 1520–1570 nm fiber-coupled laser and photodetector as depicted in Fig. 3(d) in the test setup (Supplementary Fig. 10). The pump laser is coupled with the microtroid resonator through a tapered optical fiber and the transmitted light is collected by a high-speed photodetector for analysis.

### Microassembly and testing of a series contact type RF MEMS switch

CPW line is prepared by sputter deposition and selective etching of 5 nm thick Cr and 100 nm thick Au on a silicon on insulator (SOI) substrate followed with etching of the below Si device layer using reactive ion etch (RIE). Si spacer inks are assembled on the CPW substrate. Au inks are then assembled on the Si spacer inks for electrical interconnection between patterned Au lines and top surfaces of Si spacer inks. The Si/Au composite beam ink is transferred and joined over the assembled Au inks at each end. The Si/Au composite beam ink fabrication procedure is further described at Materials and Method section in Supplementary Information. This beam is suspended at the center that can be pulled in for RF signal transmission (Supplementary Fig. 11(c)) and it is tested with the setup described in Supplementary Fig. 12 as described in Materials and Method section in Supplementary Information.

## Additional Information

**How to cite this article**: Keum, H. *et al*. Microassembly of Heterogeneous Materials using Transfer Printing and Thermal Processing. *Sci. Rep.* **6**, 29925; doi: 10.1038/srep29925 (2016).

## Supplementary Material

Supplementary Information
