# Supplementary material for: Microassembly of Heterogeneous Materials using Transfer Printing and Thermal Processing
Source: Sci Rep. 2016 Jul 18;6:29925. doi: 10.1038/srep29925 (PMC4947911; doi:10.1038/srep29925)
Supplement: Supplementary Information [file srep29925-s1.pdf]

Supplementary Information for

Microassembly of Heterogeneous Materials using Transfer Printing and Thermal Processing

Authors: Hohyun Keum[1], Zining Yang[1], Kewen Han[1], Drew E. Handler[2], Thong Nhu Nguyen[2], Jose Schutt-Aine[2], Gaurav Bahl[1] and Seok Kim[1,*]

Affiliations:

[1]Department of Mechanical Science and Engineering, University of Illinois at Urbana-Champaign, Urbana, Illinois 61801, USA

[2]Department of Electrical and Computer Engineering, University of Illinois at Urbana-Champaign, Urbana, Illinois 61801, USA

*Correspondence to: skm@illinois.edu

Supplementary Information include:

Supplementary figure 1 – Supplementary figure 13

Supplementary table 1 and Supplementary table 2

Materials and Methods

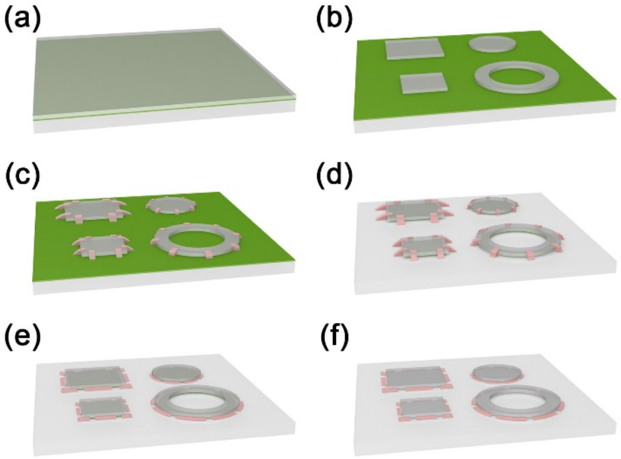

Supplementary figure 1

Schematics of silicon (Si) ink fabrication. (a) SOI wafer with desired device layer properties and 1 μm thick box oxide layer, **(b)** patterned device Si layer, **(c)** PR patterning for selective undercut etching, **(d)** Removal of exposed box oxide layer and undercut of oxide layer beneath the patterned Si, **(e)** Formation of anchors within the undercut region, **(f)** Complete removal of box oxide layer resulting in the suspended Si layer tethered by PR anchors. ****More detailed procedure is described in materials and methods section***.

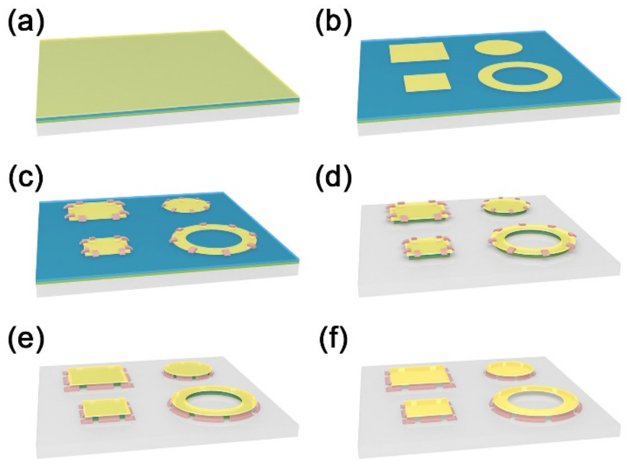

Supplementary figure 2

Schematics of gold (Au) ink fabrication. (a) ~ 1 μm thermal oxide is grown on Si wafer followed with Ti and Au deposition, **(b)** Etch back patterning of Au, **(c)** PR patterning for selective undercut etching, **(d)** Removal of exposed box oxide layer and undercut of oxide layer beneath the patterned Si, **(e)** Formation of anchors within the undercut region, **(f)** Complete removal of box oxide layer resulting in the suspended Au layer tethered by PR anchors. ****More detailed procedure is described in materials and methods section***.

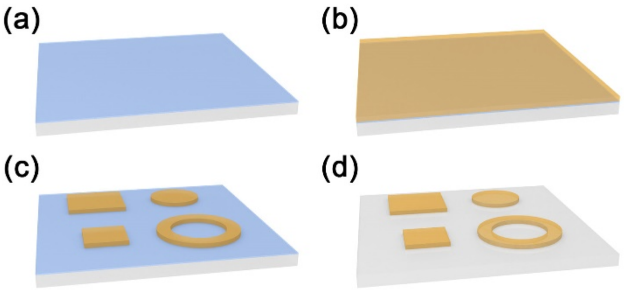

Supplementary figure 3

Schematics of SU8 ink fabrication. (a) PMMA is spin coated on Si wafer. **(b)** SU8 is spin coated and **(c)** photolithographically patterned. **(d)** Removal of PMMA layer and subsequently leaving SU8 adhered to the substrate with mere surface adhesion force. ****More detailed procedure is described in materials and methods section***.

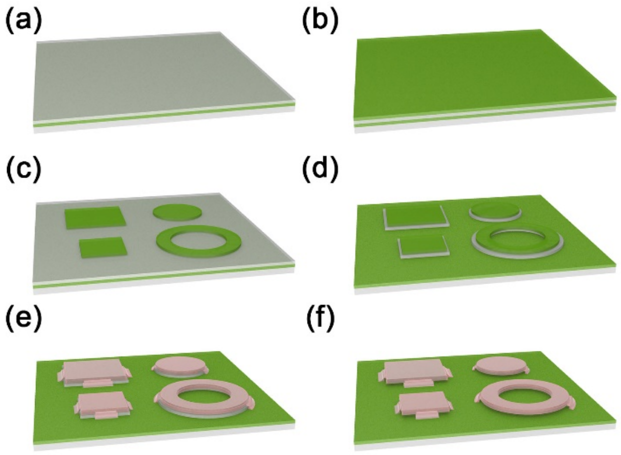

Supplementary figure 4

Schematics of silicon dioxide (SiO_2) ink fabrication. (a) SOI wafer, **(b)** Thermal oxidation of the device layer, **(c)** Patterning of thermal oxide using HF, **(d)** Removal of Si layer while ensuring there's no undercut region beneath the grown SiO_2, **(e)** PR patterning that covers the patterned SiO_2 inks while anchoring to the box oxide layer followed with **(f)** Complete removal of device Si layer. ****More detailed procedure is described in materials and methods section**.*

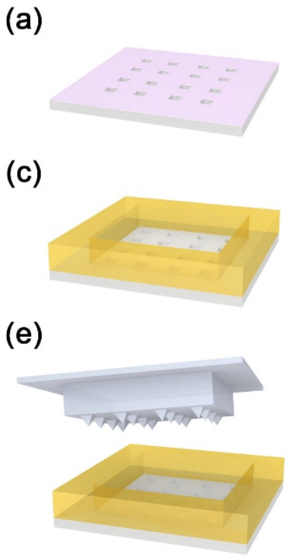

Supplementary figure 5

Schematics of microtip polydimethylsiloxane (PDMS) stamp fabrication. (a) Silicon nitride layer is deposited on Si wafer and patterned into small squares that serve as bases of the microtip. **(b)** KOH etching of the Si wafer and complete removal of the silicon nitride layer, **(c)** Formation of SU8 walls that forms a cavity, **(d)** Fill the cavity with PDMS and cure. **(e)** Peel off the fully cured microtip stamp from the negative mold. ****More detailed procedure is described in materials and methods section***.

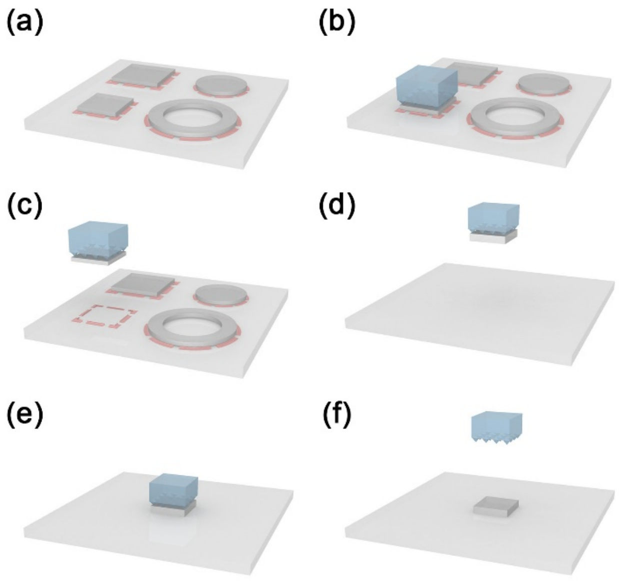

Supplementary figure 6

Procedure of transfer printing using elastomeric microtip stamp. (a) Inks are fabricated as described in **Supplementary Fig.1** through **Supplementary Fig.4**. **(b)** Elastomeric microtip stamp is brought in contact with an ink with high preload that forms fully collapsed microtips. **(c)** Rapid retrieval of the stamp leads the ink to be adhered to the microtip stamp. The previously fully collapsed microtips are fully restored that results in minimal contact area at the tips. **(d)** Deliver the ink onto target receiving substrate. **(e)** Bring the ink in contact with the receiving surface. **(f)** Slowly raise the stamp that leaves the ink on receiver substrate. ***More detailed procedure is described in materials and methods section**.

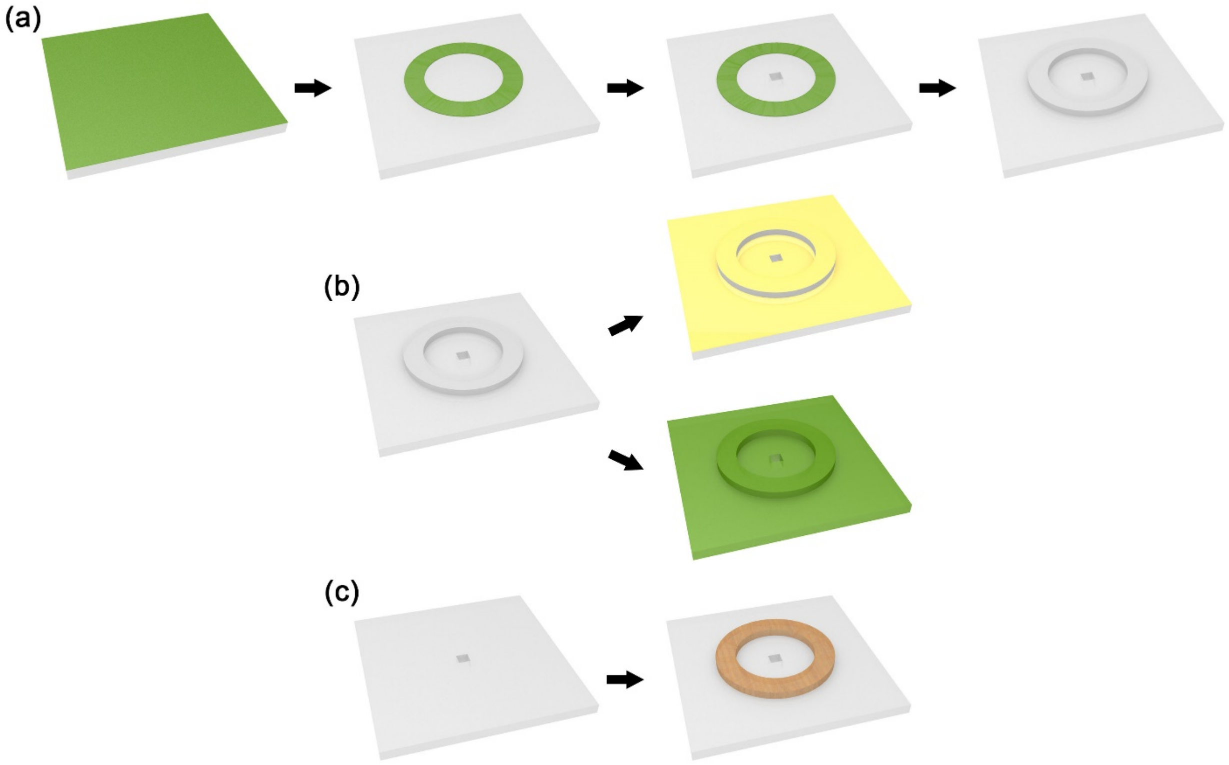

Supplementary figure 7

Schematics of various surface material blister test receiving substrate fabrication. (a) Fabrication flow of an Si receiving substrate for blister test, **(b)** Cr and Au are deposited or SiO_2 is grown on the Si receiving substrate to realize an Au or SiO_2 coated surface. **(c)** SU8 rim is patterned on an Si substrate with air inlet for SU8 specimen. ***More detailed procedure is described in materials and methods section***.

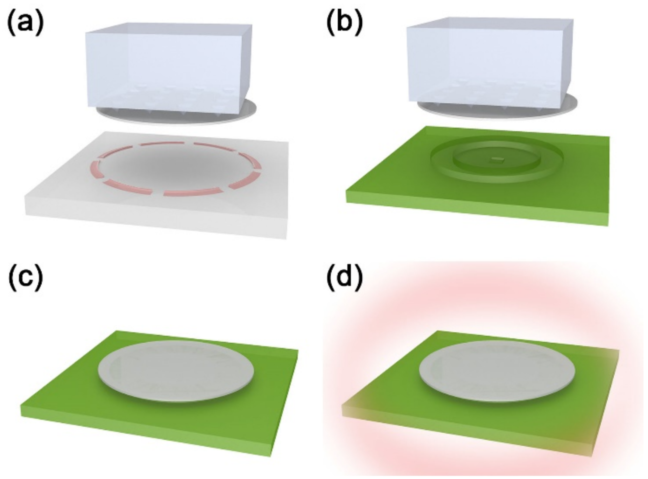

Supplementary figure 8

Schematics of blister test specimens via micro-Lego. (a) Si disc is picked up from donor substrate prepared as depicted in **Supplementary Fig.1**. **(b)** The Si disc ink is transferred onto blister test receiving specimen and **(c)** placed, and **(d)** thermally processed at proper condition for robustly joined interface. ***More detailed procedure is described in materials and methods section**.

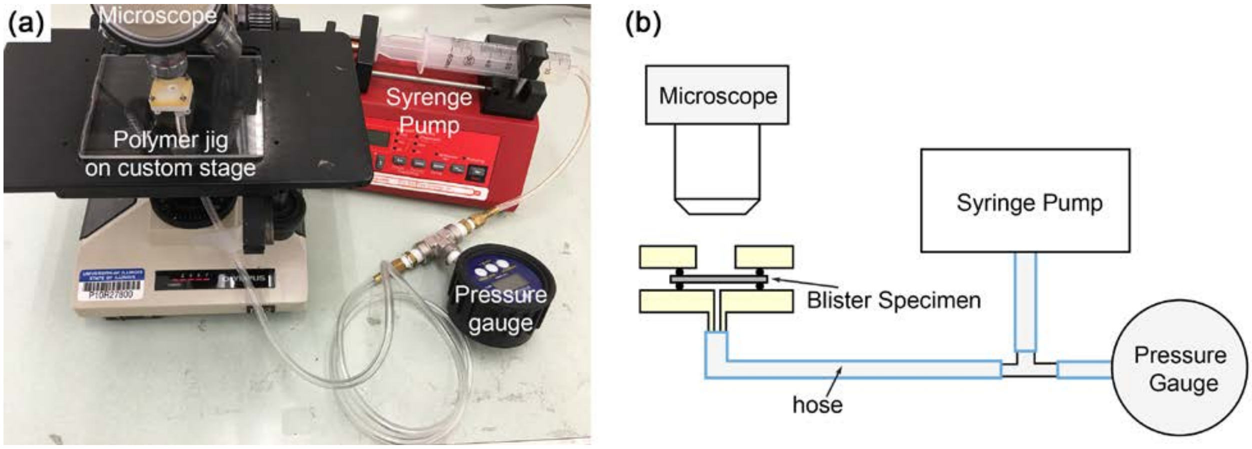

Supplementary figure 9

Optical image and schematic illustration of a blister test setup. (a) Optical image of the blister test setup, which includes polymer jig, hose, pressure gauge, pump (syringe), a custom stage and a microscope. **(b)** Cross-sectional illustration of the blister test setup

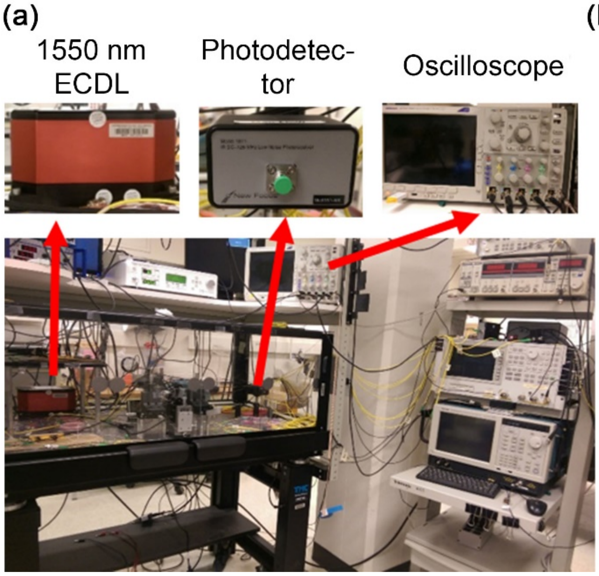

(b)

Name of Material/ Equipment	Company	Catalog Number
Tunable IR laser	Newfocus	TLB-6328
Photodetectors	Newfocus	1811-FC (Low speed 125MHz) / 1611-FC-AC (High speed 1GHz)
Optical fiber	Corning	SMF28
Oscilloscope	Tektronix	DPO 4104B-L

Supplementary figure 10

SiO_2 microtoroidal resonator measurement setup. (a) Photograph of SiO_2 microtoroidal resonator measurement setup. **(b)** Corresponding equipment models**.** A fiber-coupled IR laser (1520 to 1570 nm, ECDL = external cavity diode laser) is used as the light source for probing optical modes of the resonator. Light is evanescently coupled in and out of the optical whispering-gallery modes (WGRs) of the microtoroid through a tapered optical fiber. An oscilloscope is used to monitor the transmission while the wavelength of the laser is tuned.

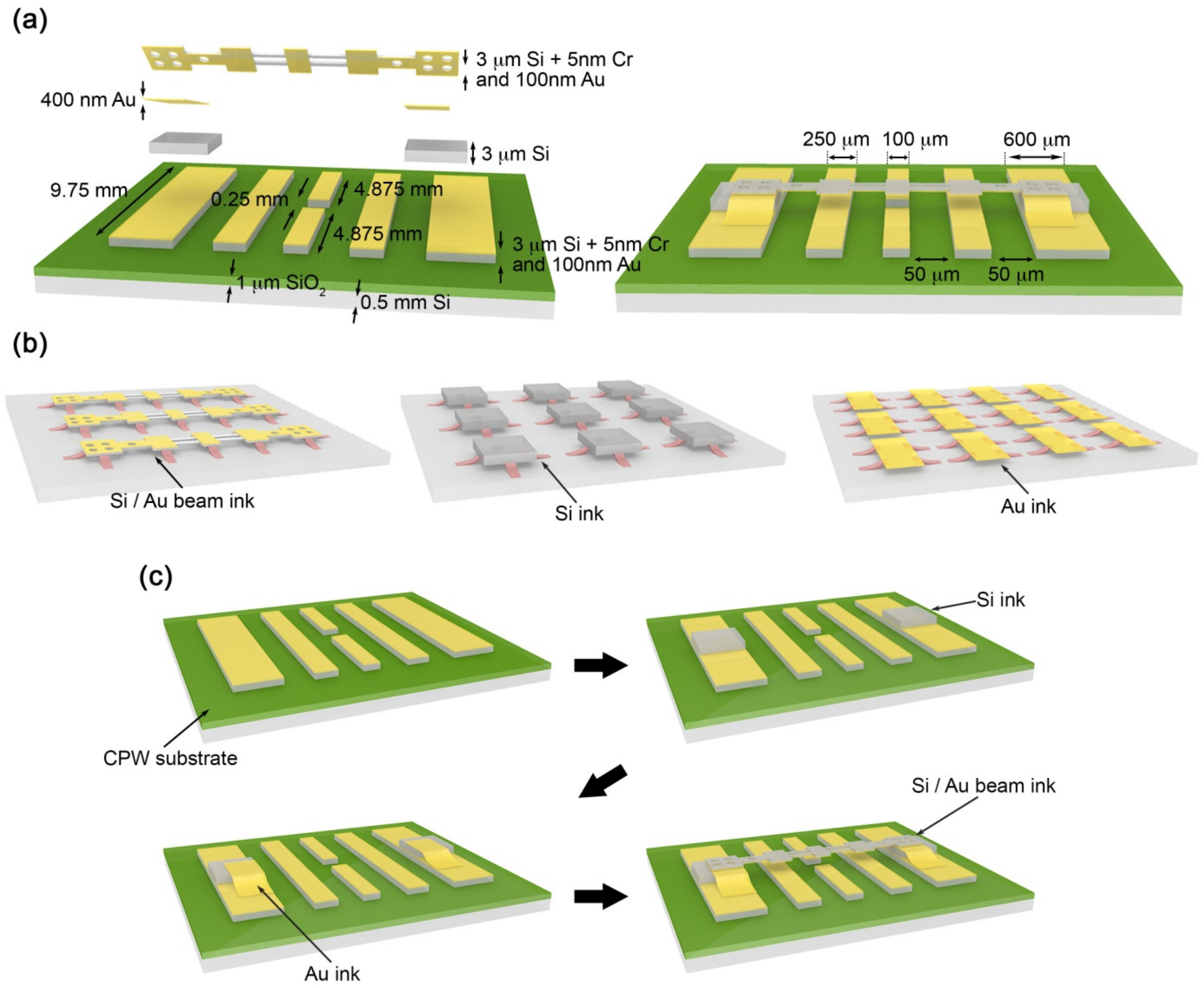

Supplementary figure 11

Schematics of RF MEMS switch fabrication via micro-Lego and respective dimensions. (a) Dimensions of CPW substrate and inks assembled for a contact type RF MEMS switch, **(b)** Si/Au beam inks, Si inks and Au inks are separately prepared on donor substrates as depicted in **Supplementary Fig.1** and **Supplementary Fig.2**. **(c)** Micro-Lego procedure of the RF MEMS switch. ***More detailed procedure is described in materials and methods section***.

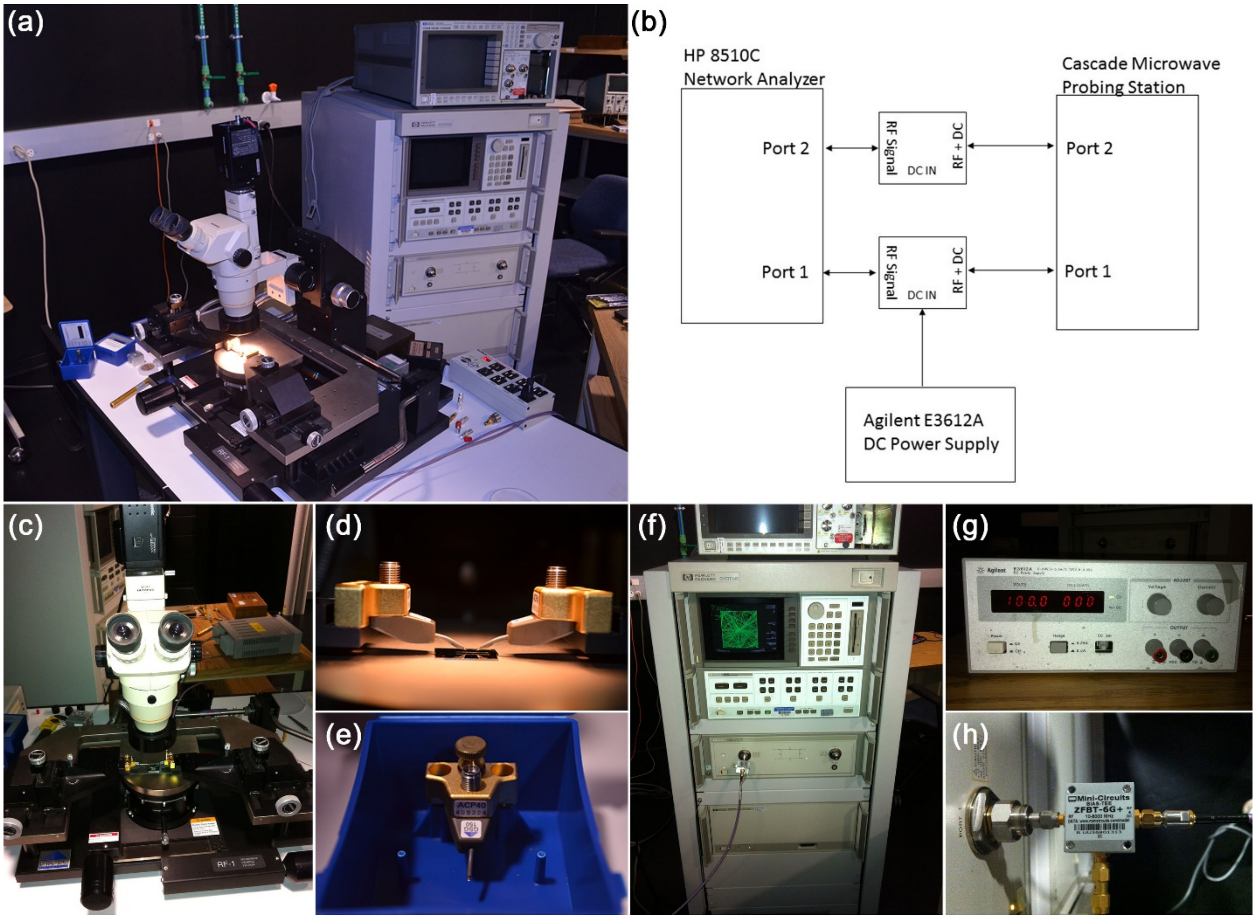

Supplementary figure 12

Optical images of RF MEMS switch measurement setup and schematics. (a) Overview of the test setup, **(b)** Schematics of the measurement setup, **(c)** Probe station, **(d)** Side view of probing the device, **(e)** Ground-signal-ground probe tip, **(f)** Network analyzer, **(g)** Power supply, **(h)** Bias-tee.

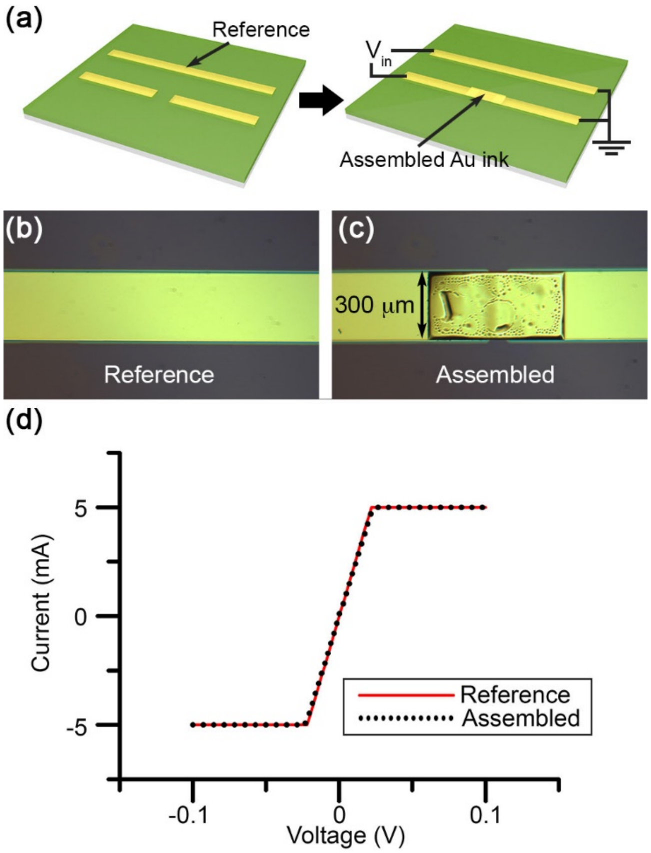

Supplementary figure 13

Total resistance of assembled Au inks. (a) Schematic of reference and assembled Au lines **(b, c)** Optical images of a reference Au line fabricated through photolithography and a connected Au line with an assembled Au ink **(d)** I-V curves of the reference and assembled Au lines.

Supplementary table 1

Detailed description of joining conditions implemented for construction of 3D structures and devices**.**

Receiving material	Ink material	Joining Condition
Si	Si	An Si ink is directly transfer printed onto a target Si surface and thermally processed in a furnace at 1000 °C for 10 min with 5 sec ramping.
Si	SiO_2	For high temperature (1000 °C) joining, a SiO_2 ink is directly printed onto a Si surface and thermally processed in a furnace at 1000 °C for 10 min with 5 sec ramping.
		For lower temperature (600 °C) joining, a SiO_2 ink is first transfer printed onto a Si substrate, which undergoes O_2 descuming process (O_2 20 sccm, 150 mTorr, 200 W, 5 min) to remove photoresist (PR) that covers the SiO_2 ink. The ink is then, transfer printed onto an activated (O_2 20 sccm, 150 mTorr, 100 W, 30 sec) Si surface. The substrate is then placed and thermally processed in a furnace at 600 °C for 10 min with 5 sec ramping.
Si	Au	The surface of the Si is cleaned with HF for removal of native oxide layer followed with transfer printing of an Au ink. The Au ink is transfer printed within short period of time after the HF treatment. The transfer printed sample is then placed in a furnace and thermally processed at 365 °C for 10 min with 5 sec ramping.
Si	SU8	An SU8 ink is directly printed on a Si surface and thermally processed in a furnace at 150 °C for 10 min with 10 min ramping.
SiO_2	Si	Si ink is directly printed onto a SiO_2 surface and thermally processed in a furnace at 1000 °C for 10 min with 5 sec ramping.
Au	Au	An Au ink is printed onto a clean Au surface with a moderate pressuring for more intimate contact.
SU8	Si, Au, SiO_2	A desired ink is printed and thermally processed in a furnace at 150 °C for 10 min with 10 min ramping.

Supplementary table 2

Thermal processing conditions for blister tests with Si inks.

Receiving Surface Materials	Joining Conditions
Si	A Si ink is transfer printed and thermally processed in a furnace at 1000 °C for 10 min with 5 sec ramping.
SiO_2	Condition 1: A Si ink is transfer printed and thermally processed in a furnace at 1000 °C for 10 min with 5 sec ramping.
	Condition 2: A receiving SiO_2 surface is activated (O_2 20 sccm, 150 mTorr, 100 W, 30 sec). Afterwards, a Si ink is transfer printed and thermally processed at 600 °C for 10 min with 5 sec ramping.
Au	Any native oxide on a Si ink is removed in a HF bath. The Si ink is transfer printed and thermally processed at 365 °C for 10 min with 5 sec ramping.
SU8	Condition 1: A Si ink is directly transfer printed and thermally processed in a furnace at 150 °C for 10 min with 10 min ramping.
	Condition 2: A SU8 receiving substrate is baked at 110 °C for 1 min followed with immersing in acetone bath for 1 min before thermal processing at 150 °C for 10 min with 10 min ramping.

Materials and Methods:

Assembly procedure of a vertical Si ring on a SU8 block in **Figure 1(e)**.

Si ring-shaped inks are prepared on a donor substrate and retrieved by a PDMS microtip stamp. On one side of the retrieved ring shaped ink, normal force is applied in horizontal direction, which causes the delamination of ink from the stamp and the ink is attached vertically on the opposite side of the stamp. Afterwards, the vertically adhering ink is transferred and joined on an SU8 block to form a 3D Si/SU8 structure.

Si ink donor substrate fabrication (**Supplementary Fig.1**).

1. Determine SOI wafer based on top Si layer thickness requirement and buried oxide (BOX) layer of 1 μm (Ultrasil): **Supplementary Fig.1(a)**
2. Pattern AZ 5214 photoresist (PR: AZ electronic materials) by first spinning at 3000 rpm for 30 seconds, followed with soft baking at 110 °C for 1 minute. Once the PR is soft baked, expose using I-line UV (Karl Suss MJB 3) for the dose of 130 mJ/cm^2 and develop using 917 MIF developer (AZ electronic materials) for approximately 20 seconds
3. Pattern Si device layer using reactive ion etching (RIE: PlasmaTherm: 40 sccm SF_6, 50 mTorr, 100 W, 3 minutes): **Supplementary Fig.1(b)**
4. Remove photoresist masking layer and pattern second mask pattern using AZ 5214 PR for selective undercut protection. Identical procedure as step 2 is used for patterning the PR.
5. Post exposure bake at 110 °C for 60 seconds: **Supplementary Fig.1(c)**
6. Place the substrate in 49% Hydrofluoric Acid (HF: Sigma-Aldrich) for 55 seconds: **Supplementary Fig.1(d)**
7. Remove photoresist and construct AZ 5214 PR anchor through identical procedure as in step 2. Since the PR anchors remain only the undercut-etched region, mask pattern is unnecessary. Flood exposure (ABM Flood Exposure Model 60) is used in this case with same dosage as in step 2: **Supplementary Fig.1(e)**
8. Post exposure bake of the PR anchors at 110 °C for 90 seconds
9. Leave the substrate for sufficient time in 49% HF bath for complete removal of BOX layer underneath the Si pattern: **Supplementary Fig.1(f)**

Au ink donor substrate fabrication (**Supplementary Fig.2**).

1. An Si wafer (University wafer) is placed inside furnace to grow ~ 1 μm of thermal oxide layer (Lindberg Hevi-Duty Lancer M-300) at 1100 °C with 6 sccm O_2 for 48 hours.
2. 5 nm of Ti and 400 nm of Au are sputter (AJA ATC ORION 8HV) deposited (20 sccm Ar, 5×10^{-3} Torr, 300 W, 1 min for Ti and 20 min for Au): **Supplementary Fig.2(a)**
3. Pattern AZ 5214 photoresist (PR: AZ electronic materials) by first spinning at 3000 rpm for 30 seconds, followed with soft baking at 110 °C for 1 minute. Once the PR is soft baked, expose using I-line UV (Karl Suss MJB 3) for the dose of 130 mJ/cm^2 and develop using 917 MIF developer (AZ electronic materials) for approximately 20 seconds.
4. Post exposure bake at 110 °C for 1 minutes.

5. Place the PR patterned substrate in Au etchant (Sigma-Aldrich) for approximately 2 minutes to pattern Au followed with PR removal: figure **Supplementary Fig.2(b)**
6. Pattern second mask pattern using AZ 5214 PR for selective undercut protection. Identical procedure as step 2 is used.
7. Post exposure bake at 110 °C for 60 seconds: **Supplementary Fig.2(c)**
8. Place the substrate in 49% Hydrofluoric Acid (HF: Sigma-Aldrich) for 55 seconds: **Supplementary Fig.2(d)**
9. Remove photoresist and construct AZ 5214 PR anchor through identical procedure as in 2. Since the PR anchors remain only the undercut-etched region, mask pattern is unnecessary. Therefore, flood exposure (ABM Flood Exposure Model 60) is used in this case with same dosage as in step 2: **Supplementary Fig.2(e)**
10. Post exposure bake of the PR anchors at 110 °C for 90 seconds.
11. Leave the substrate for sufficient time in 49% HF bath for complete removal of buried oxide and Ti sacrificial layer underneath the Au pattern: **Supplementary Fig.2(f)**

SU8 ink donor substrate fabrication (**Supplementary Fig.3**).

1. Spin coat 495 poly(methylmethacrylate) A resist with 6% in Anisole (PMMA: Microchem) at 3000 rpm and soft bake at 180 °C for 1 minutes: **Supplementary Fig.3(a)**
2. Spin coat SU8-50 (Microchem) at 3000 rpm and soft bake on hot plate for 65 °C for 6 minutes and 95 °C for 20 minutes: **Supplementary Fig.3(b)**
3. Using H-line flood exposure (ABM Flood Exposure Model 60), expose SU8-50 through a pattern mask for 200 mJ/cm^2.
4. Post exposure bake at 65 °C for 1 minutes and 95 °C for 5 minutes.
5. Develop using SU8 developer for 6-10 minutes (MicroChem): **Supplementary Fig.3(c)**
6. Submerge the substrate in acetone bath for 1 min for complete removal of PMMA sacrificial layer: **Supplementary Fig.3(d)**

SiO_2 ink donor substrate fabrication (**Supplementary Fig.4**).

1. An SOI wafer (Ultrasil) with device layer with 1.5 μm **Supplementary Fig.4(a)** is placed inside furnace to grow ~ 1 μm of thermal oxide layer (Lindberg Hevi-Duty Lancer M-300) at 1100 °C with 6 sccm O_2 for 48 hours: **Supplementary Fig.4(b)**
2. Pattern AZ 5214 photoresist (PR: AZ electronic materials) by first spinning at 3000 rpm for 30 seconds, followed with soft baking at 110 °C for 1 minute. Once the PR is soft baked, expose using I-line UV (Suss MJB 3) for the dose of 130 mJ/cm^2 and develop using 917 MIF developer (AZ electronic materials) for approximately 20 seconds.
3. Post exposure bake at 110 °C for 1 minutes.
4. Place the substrate in 49% HF (Sigma-Aldrich) for 55 seconds: **Supplementary Fig.4(c)**
5. Remove PR masking layer and Pattern AZ 5214 PR for device layer patterning following the identical procedure as in step 2. This second PR pattern is designed to be approximately 10% larger in lateral dimension than previous oxide pattern in order to protect SiO_2 layer during RIE process.

6. Pattern Si device layer using reactive ion etching (RIE: PlasmaTherm: 40 sccm SF_6, 50 mTorr, 100 W, 3 minutes) and remove PR: **Supplementary Fig.4(d)**
7. Construct AZ 5214 PR anchor using the identical procedure as in step 2. The pattern needs to completely cover patterned thermally grown SiO_2 layer as well as some anchors that extends out to buried oxide layer: **Supplementary Fig.4(e)**
8. Place the substrate in XeF_2 etcher (Xactix) for 30 cycles that runs 3 Torr XeF_2, 50 seconds per cycle for complete removal of Si device layer that served as sacrificial layer: **Supplementary Fig.4(f)**

Elastomeric microtip stamp fabrication (**Supplementary Fig.5**).

1. On {100} Si substrate (University Wafer), deposit 50 nm of Si_3N_4 using PECVD (STS systems USA Inc) with medium frequency (1960 sccm N_2, 40 sccm SiH_4, 35 sccm NH_3, 650 mTorr, 300 °C platen and 240 °C showerhead temperatures, 13.56 MHz 20W 6 seconds and 380KHz, 20W 2 seconds per cycle, total of 37 cycles).
2. Pattern AZ 5214 photoresist (PR: AZ electronic materials) by first spinning at 3000 rpm for 30 seconds, followed with soft baking at 110 °C for 1 minute. Once the PR is soft baked, expose using I-line UV (Suss MJB 3) for the dose of 130 mJ/cm^2 and develop using 917 MIF developer (AZ electronic materials) for approximately 20 seconds.
3. Post exposure bake at 110 °C for 1 min.
4. Etch Si_3N_4 using 10:1 buffered oxide etchant (BOE: Sigma-Aldrich) by submerging the substrate in the BOE bath for 150 seconds and remove AZ 5214 PR: **Supplementary Fig.5(a)**
5. Place the substrate in potassium hydroxide (KOH: Fisher Scientific), isopropyl alcohol (IPA: Fisher Scientific) and deionized water (DI) mixture bath (70g KOH, 70ml IPA and 170ml DI).
6. Place the KOH bath on 80 °C hot plate for ~ 4 hours.
7. Remove the Si_3N_4 masking layer by submerging in HF (Sigma-Aldrich) bath for 150 seconds: **Supplementary Fig.5(b)**
8. Spin coat SU8-50 (Microchem) at 3000 rpm and soft bake on hot plate for 65 °C for 6 minutes and 95 °C for 20 minutes.
9. Using H-line flood exposure (ABM Flood Exposure Model 60), expose SU8-50 through a pattern mask for 200 mJ/cm^2.
10. Post exposure bake at 65 °C for 1 minutes and 95 °C for 5 minutes.
11. Develop using SU8 developer for 6-10 minutes (MicroChem).
12. Place the substrate vertically inside desiccator and apply 3-5 droplets of (Tridencafluoro-1,1,2,2-Tetrahydrooctyl)-1-Trichlorosilane (Trichlorosilane: United Chemical Technology) inside the desiccator.
13. Leave the substrate for 1 hour in vacuum. A monolayer of trichlorosilane is deposited on the surface of the Si/SU8 substrate: **Supplementary Fig.5(c)**
14. Mix polydimethylsiloxane (PDMS: Sylguard 184, Dow Corning) pre-polymer base and cross-linking agent at 5:1 ratio.
15. Stir the mixture thoroughly and place the mixture inside a vacuum jar for 30 minutes. This step removes any micro scale bubbles that can be trapped inside the mixture.
16. Slowly pour the PDMS mixture over Si/SU8 substrate.

17. Cure the PDMS by placing the substrate inside 70 °C oven for 2 hours: **Supplementary Fig.5(d)**
18. Slowly peel off the PDMS **Supplementary Fig.5(e)** and remove the excessive PDMS using razor blade if necessary.

Transfer printing using elastomeric microtip stamp (**Supplementary figure 6**).

1. Prepare inks with desired material and dimensions following the procedures described in **supplementary Fig.1** through **Supplementary Fig.4**: **Supplementary Fig.6(a)**
2. Align microtip elastomeric stamp fabricated following figure S5 with a target ink that will be transferred.
3. Apply preload such that all microtips are fully collapsed resulting increased surface contact area: **Supplementary Fig.6(b)**
4. Rapidly retract the elastomeric microtip stamp for ink pick up. The removed preload causes the microtips to restore to original configuration that results reduced contact area between stamp and the retrieved ink: **Supplementary figure 6(c)**
5. Transfer the ink to desired receiving site: **Supplementary Fig.6(d)**
6. Contact the ink with the receiving site using marginal preload: **Supplementary Fig.6(e)**
7. Raise the stamp slowly consequently the ink is printed on substrate: **Supplementary Fig.6(f)**

Blister test receiving substrate fabrication and specimen surface materials & dimensions (**Supporting Fig.7**).

I. Si receiving substrate: **Supplementary Fig.7(a)**

1. Deposit 200 nm of SiO_2 using PECVD (PlasmaTherm) on Si wafer.
2. Pattern SiO_2 layer to ring shape by photolithography, RIE etching (Axic), 40 sccm CF_4, 200 W,35 mTorr, 20 minutes), and photoresist striping.
3. Pattern a 100 μm square via hole opening by photolithography, etch through the wafer by DRIE (STS Pegasus DRIE, C_4F_8 200 sccm, 100 mTorr, 2000 W coil power for 4 sec passivation step, SF_6 450 sccm and O_2 45 sccm, 100 mTorr, 2800 W coil power, 40 W platen power for 7 sec etch step), and strip photoresist.
4. Etch silicon using DRIE with oxide mask on (STS Pegasus DRIE).
5. Dip the silicon substrate into hydrofluoric acid to remove SiO_2 mask.
6. Cleavage the wafer into chips.

II. Au and SiO_2 receiving substrate: **Supplementary Fig.7(b)**

1. Both Au and SiO_2 begin with a Si receiving substrate prepared in figure S7(A).
2. For Au receiving sample, 400 nm Au is sputter (AJA ATC ORION 8HV) deposited (20 sccm Ar, 5×10^{-3} Torr, 300 W, 20 min for Au).
3. For SiO_2 receiving substrate, the Si receiving substrate is placed inside furnace (Lindberg Hevi-Duty Lancer M-300) at 1100 °C with 6 sccm O_2 for 24 hours to thermally grow 700 nm of SiO_2.

III. SU8 receiving substrate: **Supplementary Fig.7(c)**

1. Via hole is constructed using DRIE (STS Pegasus DRIE).

2. Spin coat SU8-50 (Microchem) at 3000 rpm and soft bake on hot plate for 65 °C for 6 minutes and 95 °C for 20 minutes.
3. Using H-line flood exposure (ABM Flood Exposure Model 60), expose SU8-50 through a pattern mask for 200 mJ/cm^2.
4. Post exposure bake at 65 °C for 1 minutes and 95 °C for 5 minutes.
5. Develop using SU8 developer for 6-10 minutes (MicroChem).

IV. Summary of joining rim dimensions and joining surfaces

Material	Joining Rim Inner Diameter	Joining Rim Outer Diameter	Joining Surface
Si receiving surface	600 μm	800 μm	Si Rim
Au-coated receiving surface	600 μm	800 μm	400 nm thick Au
SiO_2-grown receiving surface	600 μm	800 μm	700 nm thick Oxide
SU8-patterned receiving surface	600 μm	800 μm	40 μm thick SU8-50

Blister test specimen assembly via micro-Lego (**Supplementary Fig.8**).

1. Prepare 3 μm thick, 900 μm diameter Si ink following the procedure described in figure S1 and microtip stamp as shown in **Supplementary Fig.5**
2. Bring the Si ink and PDMS stamp together with high preload and rapidly raise the stamp to retrieve the Si disc ink: **Supplementary Fig.8(a)**
3. Transfer the Si ink onto receiving substrate prepared in **Supplementary Fig.7: Supplementary Fig.8(b)**
4. Print the ink with precision: **Supplementary Fig.8(c)**
5. Thermally process the specimen for hermetic joining throughout the contact area: **Supplementary Fig.8(d)**

Cr/Au deposited Si flexure beam ink preparation for the RF MEMS switch (**Supplementary Fig. 11(a)**).

For the Cr/Au deposited Si flexure beam is prepared by first depositing 5 nm Cr and 100 nm Au using sputter (AJA ATC ORION 8HV: 20 sccm Ar, 5×10^{-3} Torr, 300 W, 1 min for Cr and 5 min for Au) followed with etching using Cr and Au etchants (Microchem) to form electrical lines in flexure beam. The device layer of SOI wafer is patterned using RIE (PlasmaTherm: 40 sccm SF_6, 50 mTorr, 100W, 3 minutes). Subsequent step is identical with fabrication of Si ink depicted in **Supplementary Fig. 1**.

RF MEMS switch FEA simulation.

The RF MEMS switch beam actuation voltage is simulated through FEA. As shown here, the center signal line is deflected slightly higher than 3 μm upon 450 Pa, which is the spacing between the beam and CPW lines. Such pressure can be converted to required voltage by assuming parallel plate electrostatic actuation as shown

$$F = \frac{\varepsilon A V^2}{2d^2}$$ (**Supplementary Equation 1**)

where ε, A, V and d represent permittivity, area, voltage bias and the distance between two parallel plates, respectively. Based on **Supplementary Equation 1**, 25 V translates into 450 Pa, which is the sufficient amount of force that allows for the beam deflection and metal-to-metal contact between the beam and the central signal line as confirmed by FEA results.

RF MEMS switch setup description (**Supplementary Fig. 12**).

To take S-parameter measurements of the RF MEMS switch, a network analyzer (HP8510C) was used in conjunction with a microwave probing station (Cascade). The probe tips, which use a ground-signal-ground (GSG: Air Coplanar™) configuration, and the GSG lines on the actual switch both had a 150 μm pitch. In order to bias the RF MEMS switch, an DC Power Supply (Agilent E3612A) was used. The “RF Data” connection for the bias tee is connected to Port 1 of the network analyzer. Then, the “RF Data + DC Bias” connection for the bias tee is connected to the probe tips on the microwave probe station. The DC connection on the bias tee is connected to the DC power supply.

The Mini-Circuits bias tee provided a convenient connection point so that the DC ground was connected to the signal ground. Alternatively, the internal bias tee on the network analyzer could also have been used. In order to actually apply the DC bias to the beam, individual probes were also connected to the DC power supply. These probes then made contact with the Au lines that the beam was electrically connected to. This supplied the DC voltage differential necessary to actuate the switch.
